# Case Report: Diagnosis and treatment of congenital right atrial dissection associated with atrial aneurysm

**DOI:** 10.3389/fcvm.2025.1463926

**Published:** 2025-03-20

**Authors:** Yuan-yuan Zhang, Chi-heng Zhou, Qun-shen Shen

**Affiliations:** Congenital Heart Disease Center, Wuhan Asia Heart Hospital, Wuhan, China

**Keywords:** congenital right atrial dissection, right atrial aneurysm, case report, atrial aneurysm, right atrial enlargement

## Abstract

Spontaneous atrial dissection and atrial aneurysm are extremely rare conditions. This may be the first reported case diagnosed in the fetal period and successfully treated with surgery under extracorporeal circulation in infancy. Although the infant in this case underwent surgery successfully, recovered, and was discharged, the optimal timing for surgical intervention in such cases remains unclear. This report aims to provide a treatment reference for similar cases.

## Introduction

Atrial dissection is an extremely rare condition, primarily related to surgical procedures such as mitral or aortic valve surgery, percutaneous coronary interventional treatment, and catheter-based or surgical arrhythmia ablation. It is more commonly observed in the left atrium (1–3). Congenital atrial dissection is even rarer and is characterized by isolated right atrial enlargement of unknown cause (4). This article reports a case of right atrial dissection detected during a prenatal examination and successfully treated by surgery after birth.

## Case introduction

**Figure 1:**
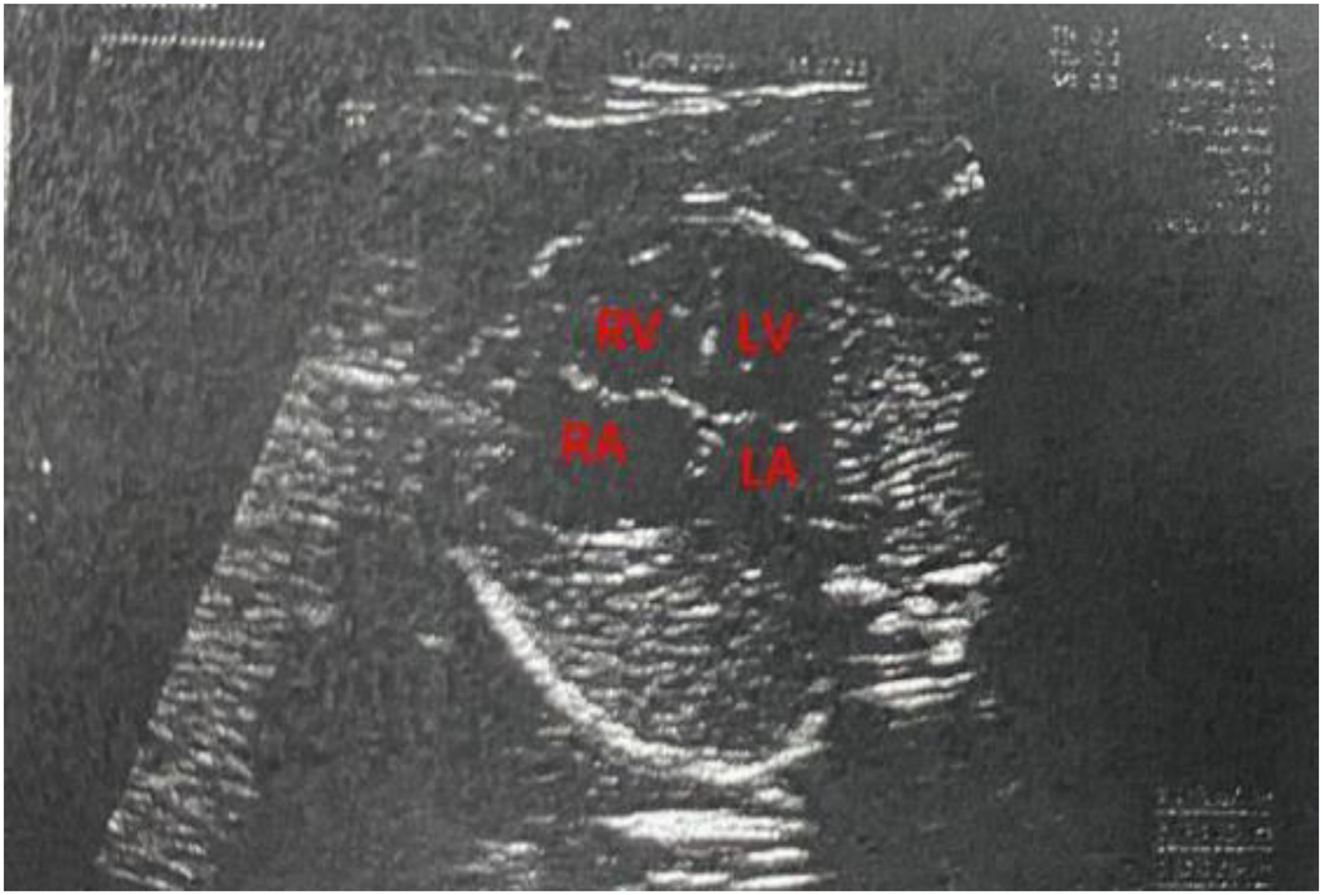
A fetal echocardiogram at 25 weeks of pregnancy. RV, right ventricle; RA, right atrium; LV, left ventricle; LA, left atrium.

**Figure 2:**
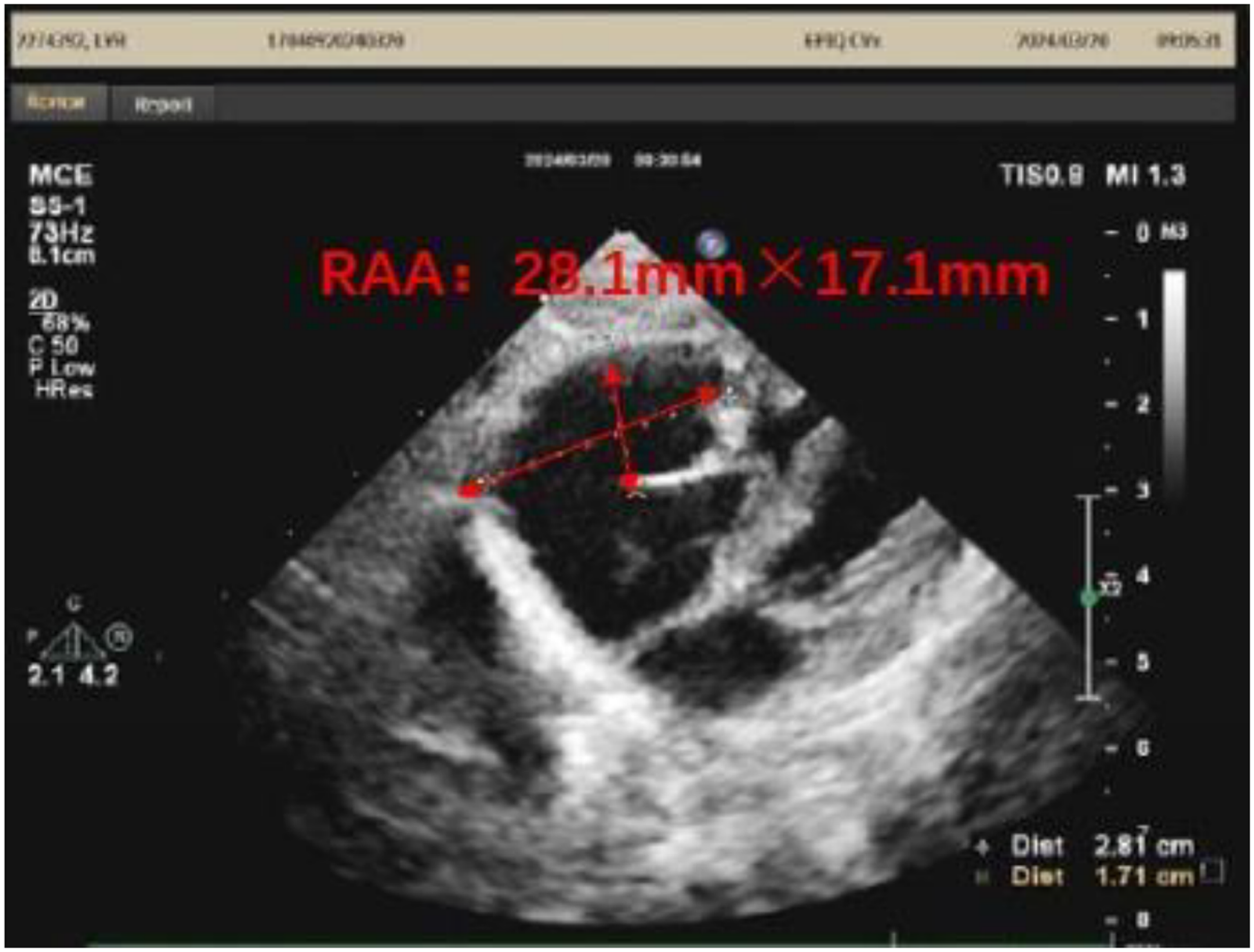
A transthoracic echocardiogram of the infant at 1 month of age. RAA, right atrial aneurysm.

**Figure 3:**
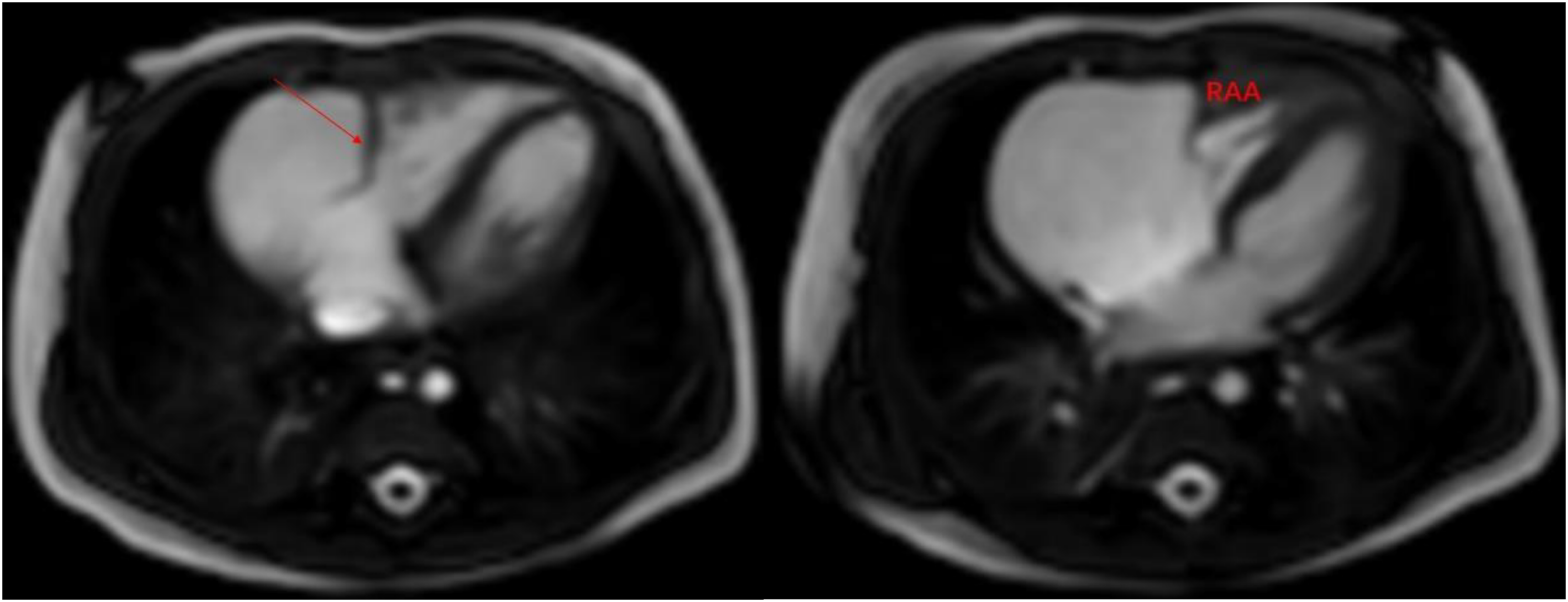
Cardiac magnetic resonance imaging: the arrow shows the atrial dissection. RAA, right atrial aneurysm.

**Figure 4:**
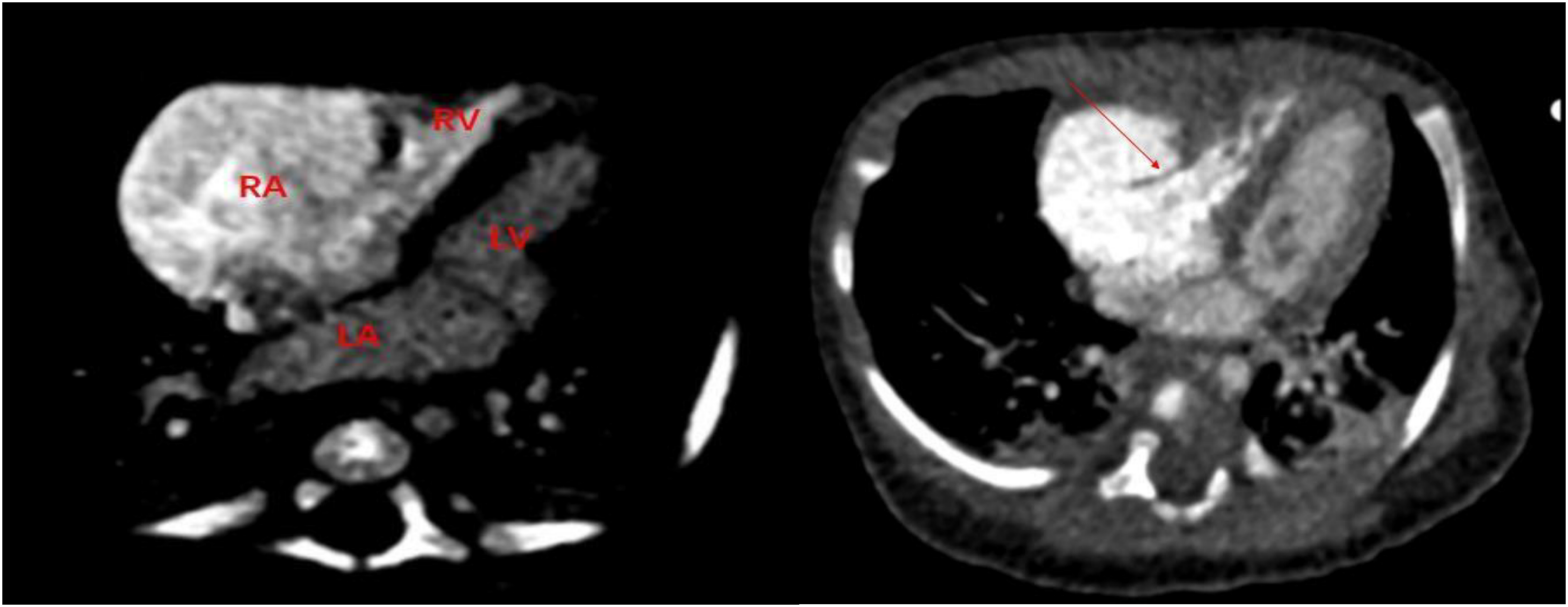
Computed tomography shows the large right atrium and atrial dissection.

A 1-month-old infant was brought to our hospital with right atrial enlargement. Fetal echocardiography at 25 weeks of gestation initially detected an enlarged right atrium (1.67 cm × 1.25 cm) (Figure 1). Subsequent prenatal follow-ups showed progressive enlargement (27 weeks: 2.48 cm × 1.35 cm; 31 weeks: 2.47 cm × 2.04 cm; 33 weeks: 3.45 cm × 2.40 cm; 36 weeks: 3.40 cm × 2.50 cm). After consultation with the obstetrics and gynecology department and with the consent of the family, the pregnancy was terminated at 36 weeks. Postnatally, the infant was not promptly treated by a cardiologist due to jaundice. The infant was brought to our hospital 1 month after birth. Physical examination was unremarkable. Laboratory tests showed an NT-ProBNP level of 1,433 pg/ml, with normal liver function, kidney function, myocardial enzyme spectrum, electrolytes, tumor markers, and routine blood count. A 24-h dynamic electrocardiogram recorded a total heart rate of 221,880 beats, with a maximum of 196 bpm (during blood sampling), a minimum of 89 bpm (during deep sleep), and an average heart rate of 153 bpm. Sinus rhythm was maintained with no arrhythmic events. Transthoracic echocardiography showed significant right atrial enlargement with localized abnormal expansion and severe tricuspid insufficiency. The right atrium and right atrial appendage were significantly dilated, and a 2.8 cm × 1.7 cm tumor-like structure was visible in the anterior right atrium, protruding outward with reduced mobility. Intima separation echo was visible locally, suggesting a high possibility of atrial aneurysm and atrial dissection. The tricuspid valve echo appeared normal, with a normal attachment point, an inner diameter of the valve ring of 1.0 cm, and a regurgitant jet with a velocity of 2.4 m/s visible in the right atrium during systole (Figure 2). Cardiac magnetic resonance imaging showed an atrial aneurysm in the anterior and upper outer part of the right atrium, measuring 28.1 mm × 27.4 mm × 35.6 mm, with a right atrial cross-section of 18.7 mm × 15.4 mm. The endocardial sheet at the top of the right atrium extended downward, connecting to the anterior leaflet of the tricuspid valve. During systole, the aneurysm compressed the free wall of the right ventricle, causing severe regurgitation. Congenital right atrial dissection with a large atrial aneurysm was suspected (Figure 3). Computed tomography showed that the right atrium and atrial appendage were significantly enlarged, and the possibility of a right atrial aneurysm combined with dissection was considered (Figure 4). In summary, the infant had congenital atrial dissection combined with atrial aneurysm and tricuspid regurgitation. Given the infant’s poor development (length 54 cm, weight 4.3 kg) and heart failure, the infant underwent atrial dissection and atrial aneurysm resection at the age of 3 months (height 61 cm, weight 7.0 kg) after the parents gave their consent.

**Figure 5:**
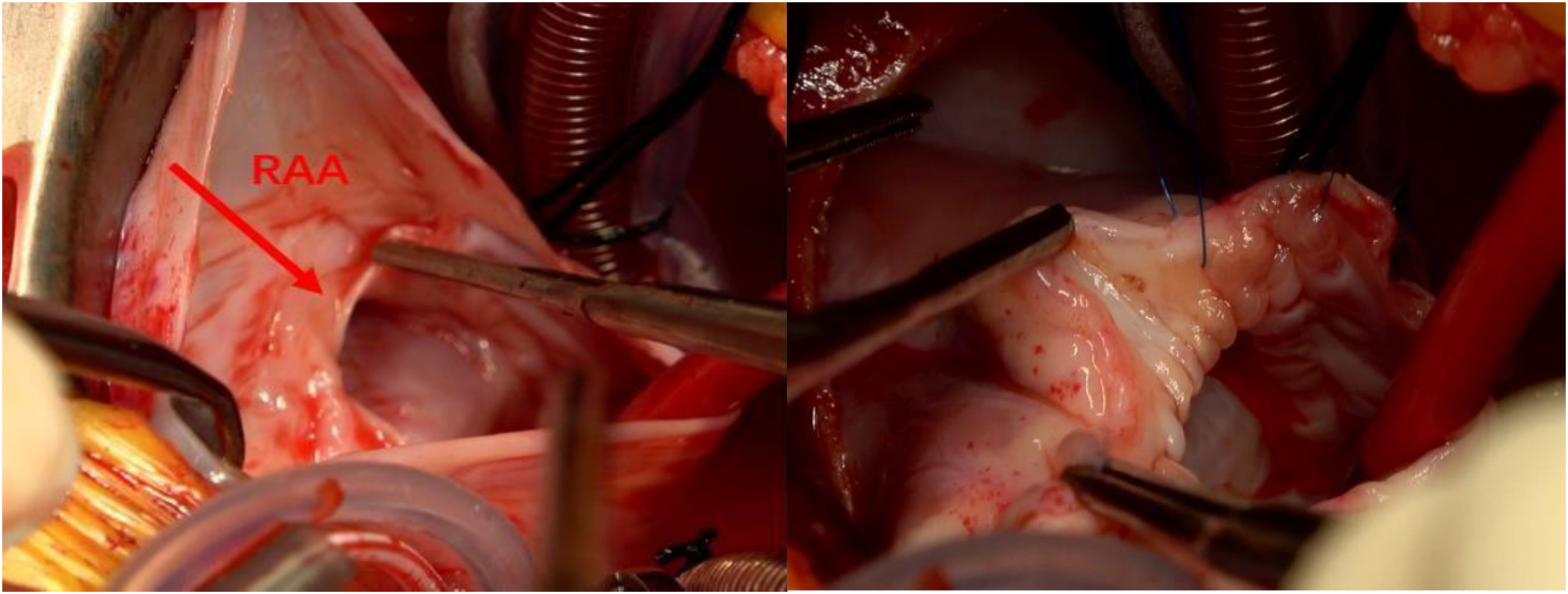
Atrial aneurysm and atrial dissection are observed during surgery.

**Figure 6:**
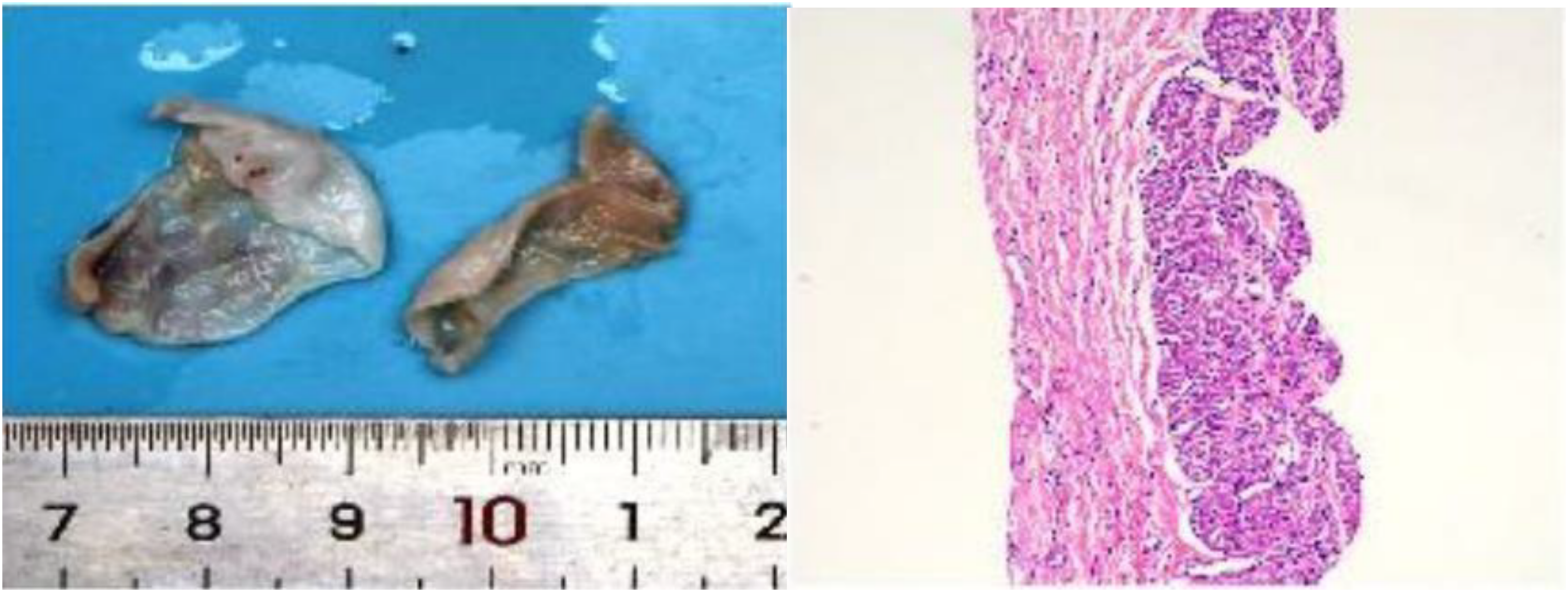
Resected right atrial tissue and pathological examination (left: right atrial tissue, two pieces of gray-white tissue, 3.5 cm × 3 cm × 2 cm in size; right: hematoxylin and eosin staining 10 × 10 microscopy shows fibrous tissue and myocardial cells).

**Figure 7:**
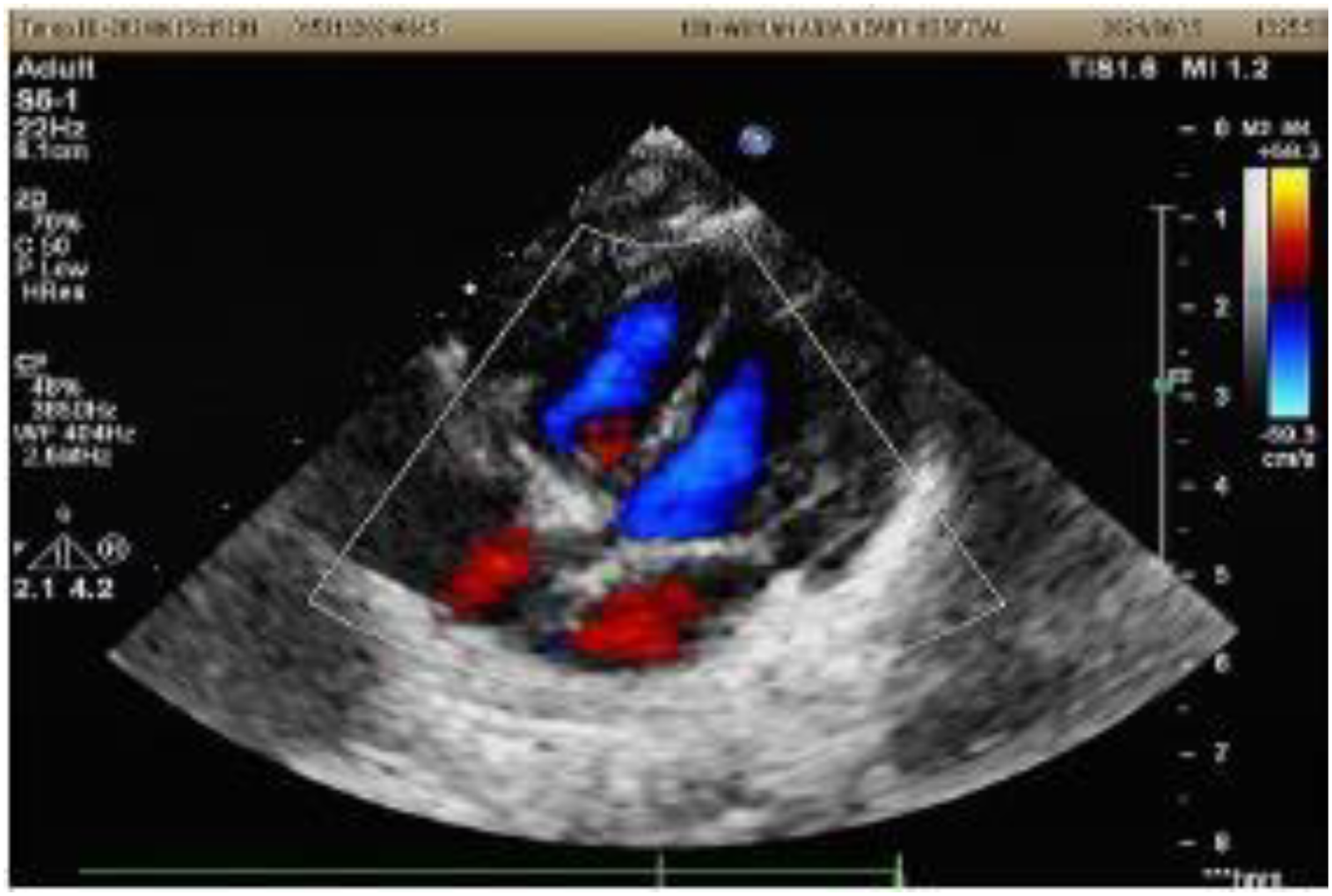
Before discharge from the hospital, the right atrial structure is essentially normal.

During surgery, the heart was enlarged, mainly in the right atrium. The heart was accessed through the ascending aorta and the right atrium, revealing a right atrial dissection progressing from the free wall to the right atrioventricular groove. No abnormalities were detected in the right atrial appendage. The tricuspid valve ring was enlarged, with moderate regurgitation. An atrial dissection combined with an atrial wall aneurysm and tricuspid valve regurgitation was diagnosed (Figure 5). During surgery, the dissected atrial wall was excised, and the right atrioventricular groove and tricuspid valve ring were reconstructed. After the patient’s heart was restarted, transesophageal echocardiography revealed mild tricuspid valve regurgitation and slow forward blood flow. The resected right atrial tissue was stained with hematoxylin and eosin (10 × 10), confirming the presence of fibrous tissue and myocardial cells, consistent with the diagnosis of atrial mural aneurysm (Figure 6). Postoperatively, the patient remained in the intensive care unit with assisted ventilation and received symptomatic treatment, such as anti-infective therapy, myocardial nutrition, and maintenance of internal environment homeostasis. After 3 days, the patient’s condition stabilized, and he was transferred to the general ward, where he continued to receive the same treatment. He was discharged from the hospital on the 13th postoperative day. A transthoracic echocardiogram before discharge showed a right atrial anteroposterior diameter of 1.6 cm with no evidence of tricuspid regurgitation (Figure 7).

## Discussion

This article reports a case of congenital right atrial dissection combined with an atrial wall aneurysm in a 3-month-old infant. According to the previous literature, reported cases of right atrial dissection are very rare and are typically related to iatrogenic operations (5). Another documented case of congenital right atrial dissection with an atrial wall aneurysm involved a 4-year-old child. In that case, an enlarged right atrium was first detected during a prenatal examination at 36 weeks of gestation. After birth, the child experienced no discomfort and was followed up regularly. Finally, due to the potential risk posed by the atrial wall aneurysm, the child underwent surgical resection of the atrial dissection at the age of 4 years. During the postoperative follow-up, the child's development was comparable to that of his peers (6). Previous reports suggest that catheter-related atrial dissection can be managed conservatively when hemodynamic changes are not life-threatening (7, 8). With advancements in prenatal screening and fetal echocardiography, atrial enlargement can now be detected earlier, as shown in this case where fetal right atrial enlargement was first observed at 25 weeks of gestation.

The main challenge of this case was determining the optimal timing of surgery for spontaneous atrial dissection. The mother's prenatal check-up at 36 weeks of pregnancy suggested the possibility of fetal atrial dissection and atrial aneurysm. A postnatal examination showed severe tricuspid valve regurgitation and heart failure, making the need for surgery clear. However, due to limited clinical experience in such cases and the infant's premature birth, young age, and low weight, the surgical risk was extremely high. After discussion within the surgical team and with the consent of the infant’s guardian, the decision was made to postpone the surgery temporarily and to closely monitor the infant. Surgery was then scheduled for 3 months of age.

The infant’s preoperative transthoracic echocardiogram revealed significant right atrial enlargement, an enlarged tricuspid valve annulus, and a normal tricuspid valve attachment point and echo. Cardiac magnetic resonance imaging showed that the inner membrane of the right atrium was connected to the anterior tricuspid valve leaflet and contracted. The atrial aneurysm compressed the right ventricular free wall, exacerbating tricuspid regurgitation. During surgery, the atrial malformation was corrected, and the tricuspid valve annulus was repaired. Postoperative transthoracic echocardiography showed a significant reduction in right atrial size and no tricuspid valve regurgitation. Based on the structural changes observed on pre- and postoperative echocardiography, the tricuspid regurgitation was likely caused by annular dilatation due to the atrial aneurysm.

However, there were limitations in the diagnosis and differential diagnosis of this case. The infant's atrial enlargement needed to be differentiated from conditions such as dilated cardiomyopathy and right atrial appendage aneurysm. According to the literature (9), the diagnosis of right atrial appendage aneurysm is based on (1) disproportionate dilation of the right atrium and (2) exclusion of other causes of atrial enlargement, such as Ebstein anomaly, tricuspid stenosis, pulmonary hypertension, and pulmonary embolism. In this case, relevant preoperative examinations and intraoperative exploration revealed that the dilation was mainly located in the anterior and lateral right atrium, with no abnormalities in the right atrial appendage. In addition, atrial dissection should be differentiated from Ehlers–Danlos syndrome. A detailed inquiry into the infant's family history found no similar cases, and genetic screening was recommended to clarify the diagnosis. However, the parents refused due to financial constraints. Despite the infant’s young age, the severity of the tricuspid regurgitation, heart failure, and critical condition required early correction of the atrial malformation. In the future, with the advancements in medicine and research into such conditions, more cost-effective, simple, efficient, and accurate diagnostic and treatment options will become available.

## Data Availability

The original contributions presented in the study are included in the article/Supplementary Material, further inquiries can be directed to the corresponding author.
